# Nonoperative, open reduction and internal fixation or primary arthrodesis in the treatment of Lisfranc injuries: a prospective, randomized, multicenter trial – study protocol

**DOI:** 10.1186/s12891-018-2222-4

**Published:** 2018-08-21

**Authors:** Ville T. Ponkilainen, Ville M. Mattila, Heikki-Jussi Laine, Antti Paakkala, Heikki M. Mäenpää, Heidi H. Haapasalo

**Affiliations:** 1 University of Tampere, School of Medicine, 33520 Tampere, Finland; 2 Department of Orthopaedics and Traumatology, Tampere University Hospital, Teiskontie 35, PL2000, 33521 Tampere, Finland; 3 COXA Hospital for Joint Replacement, Biokatu 6, 33520 Tampere, Finland; 4 Department of Radiology, Tampere University Hospital, Teiskontie 35, PL2000, 33521 Tampere, Finland

**Keywords:** Lisfranc, Conservative treatment, Operative treatment, ORIF, Arthrodesis

## Abstract

**Background:**

Lisfranc injuries are known to be rare and often overlooked injuries that can cause long-term disability and pain when missed or treated incorrectly. The wide variety of Lisfranc injuries ranges from subtle ligament distensions to open fracture dislocations. The treatment of Lisfranc joint injuries is still controversial and very little is known about what types of injury can be treated nonoperatively. The current literature provides only two randomized studies on dislocated Lisfranc injuries. These studies have shown that primary arthrodesis (PA) leads to a similar or better outcome and results in fewer secondary operations when compared with open reduction and internal fixation (ORIF) in ligamentous injuries. There have been no previous randomized studies of the nonoperative versus operative treatment of Lisfranc injuries. Therefore, the purpose of this study is to compare the operative and nonoperative treatment of non-dislocated Lisfranc injuries and to compare the ORIF and PA treatment of dislocated Lisfranc injuries.

**Methods:**

This study is a prospective, randomized, national multi-center trial. The trial comprises two strata: Stratum I compares cast-immobilization versus open reduction and internal fixation (ORIF) treatment of non-dislocated Lisfranc joint injuries. Stratum II compares PA versus ORIF in the treatment of dislocated injuries of the Lisfranc joint. The main hypothesis of stratum I is that the nonoperative treatment of non-dislocated Lisfranc injuries achieves a similar outcome compared with operative treatment (ORIF). The hypothesis of stratum II is that PA of dislocated Lisfranc injuries yields a similar functional outcome compared with ORIF, but that PA results in fewer secondary operations than ORIF. The main outcome measure is the American Orthopaedic Foot and Ankle Society (AOFAS) Midfoot score and the secondary outcome measures are Visual-Analogue-Scale Foot and Ankle (VAS-FA), Visual-Analogue-Scale (VAS), rate of secondary operations and other treatment-related complications. The results will be analyzed after the 2-year follow-up period.

**Discussion:**

This publication presents a prospective, randomized, national multi-center trial study protocol. It provides details of patient flow, randomization, aftercare and methods of analysis of the material and ways to present and publish the results.

**Trial registration:**

ClinicalTrials.gov identifier: NCT02953067 24.10.2016.

## Background

Named after Jaques Lisfranc, an eighteenth century surgeon who performed the first foot amputations at the tarsometatarsal (TMT) joint, the Lisfranc joint is an anatomic area where a broad spectrum of injuries from subtle distensions to open fracture dislocations occur [1, 2]. The incidence of Lisfranc injuries is estimated to be 1/55000/year and they are believed to account for 0.2% of all fractures [3, 4]. These figures have, however, been challenged as up to 24% of Lisfranc injuries are either misdiagnosed or overlooked during initial evaluation [5–7]. Injuries to the Lisfranc joint occur most often during the third decade of life and men are 2 to 4 times more likely to suffer from these injuries than women [8]. Lisfranc injuries are caused either by direct or indirect forces to the foot [9]. Indirect injuries are more common and occur during bending or twisting movements applied to the midfoot [9]. Injuries caused by direct forces are often induced by a heavy object falling on top of the foot or by crush injuries, such as in motor vehicle accidents [6, 7]. A wide spectrum of injuries to the TMT and interrelated joints have been recognized, and range from severely dislocated high-energy open injuries to minor midfoot sprains suffered during sports activities [10–12].

An untreated or inadequately treated Lisfranc injury results in multiple late complications, the severity of which depends on the severity of the primary injury [13]. The most common complications are painful instability of the joint, malformation and arthritis [5]. All these complications can lead to remarkable dysfunction and foot pain [5]. Secondary arthrodesis may be used to treat these injuries, but the outcome is poorer the longer the treatment is delayed [14–16]. Therefore, the initial recognition of these injuries is a crucial step in ensuring optimal treatment is provided.

### Diagnosis and treatment

Fractures of the Lisfranc joint are known to be rare and are often overlooked [7, 17–19]. Approximately 20 to 24% of these fractures are missed at initial evaluation [5, 7]. High-energy injuries are often the most obvious due to traumatic history and very apparent clinical findings [20]. Low-energy injuries, however, are harder to detect because of less traumatic history and less apparent clinical findings [21]. Typical clinical findings of fracture of the Lisfranc joint are a swollen midfoot, tenderness and pain in the midfoot during passive movements and weight-bearing [22], plantar ecchymosis [23] and an extended space between the first and second toe seen in x-ray radiographs that is also known as the ‘gap’ sign [24].

Although sensitivity is relatively low when compared with CT-imaging, primary diagnosis of Lisfranc injuries is usually based on plain x-ray imaging [7]. False-negative findings on x-ray radiographs may be the result of weight-bearing not tolerated due to pain [6]. A typical finding ‘fleck sign’ in plain x-ray radiographs, an avulsion of intra-articular bone, is estimated to be detectable in 90% of cases where the dislocation between the first and second metatarsal is greater than 4 mm [5]. As the radiographic findings of Lisfranc injuries can be subtle, CT is an important imaging modality in detecting these injuries, and furthermore serves as a useful tool for preoperative planning [25, 26]. Although the current literature introduces classifications that provide general characteristics for Lisfranc injuries, none of the classifications are useful in predicting treatment or outcome of a Lisfranc injury [27]. Moreover, the current literature fails to offer a classification based on computed tomography.

Due to the diversity of injuries, there is no single evidence-based policy for treating all Lisfranc injuries in a similar manner [28]. Nowadays, there is strong consensus that in dislocated injuries it is crucial to achieve exact anatomic reduction and stable internal fixation, which is best obtained with open reduction and screw fixation (ORIF) [5, 29]. However, even after appropriate treatment with ORIF, up to 40 to 94% of patients will develop post-traumatic arthritis [5, 13, 30, 31], necessitating conversion to an arthrodesis to relieve pain [14–16]. To prevent the need for secondary operations and the development of post-traumatic arthritis, primary arthrodesis (PA) is suggested [30, 32–34]. The treatment of non-dislocated injuries, in turn, is controversial [29, 35–38]. Some stable injuries might need activity modification only, but surgery is often recommended for even minimally displaced injuries [5, 29]. There is general agreement, however, that poor functional results are commonly correlated with a delay in diagnosis or the inadequate treatment of unstable or dislocated injuries [19, 27].

Fixation with screws is the primary fixation technique used to treat dislocated Lisfranc injuries [13, 31]. K-wire fixation [5, 12, 39, 40] and screw fixation [13, 22, 41, 42] are both controversial, but the higher failure rates associated with K-wire fixation have led to an increase in screw fixation [13, 43, 44]. Another fixation technique, dorsal plate fixation, has been reported to produce similar results as ORIF [45]. An advantage of dorsal plate fixation is that the plate causes no damage to the articular surface. However, soft-tissue irritation may be more prevalent, and second surgery is often needed to remove the plates [45].

There is, however, no general agreement on what is the correct nonoperative protocol for treating non-dislocated Lisfranc injuries. In their review, Myerson and Cerrato [11] concluded that if the foot remains stable in weight-bearing radiographs 2 weeks after the injury, the injury can be treated with immobilization in a boot and weight-bearing is permitted as tolerated until the boot is removed at six to eight weeks. In the study by Nunley & Vertullo [29], stable injuries were treated nonoperatively. Furthermore, it was suggested that treatment begin with a non-weight-bearing cast for 6 weeks. If the patient is painless at 6 weeks, treatment should continue with a gradual return to normal function with a weight-bearing orthosis for the following 4 weeks.

The commonly used postoperative protocol is nearly identical to nonoperative treatment. In their study, Ly & Coetzee [30] used a short leg splint for 2 weeks followed by a short leg cast for four to six weeks. The patients advanced to full weight-bearing during the following 4 weeks while wearing a prefabricated fracture boot. In the study by Henning et al. [33], weight-bearing began at three months with a controlled ankle motion walker.

Interestingly, there are several opinions about postoperative implant removal. Some studies suggest routine screw removal at 8 or 12 weeks [31, 33, 46, 47], while others prefer routine removal only after the recovery is complete or only if the screws cause irritation or pain [48–50]. Ahmad and Jones [51] have suggested the use of bioabsorbable screws to remove the need for screw removal. In addition, bioabsorbable screws achieve similar functional results compared with metal screws.

### Evaluation of treatment

Most of the previous studies have used Patient Reported Outcome Measures (PROMs) to evaluate treatment. The most common PROM used in Lisfranc injury studies is the American Orthopaedic Foot and Ankle Society Midfoot Score (AOFAS) [13, 28, 30, 38, 47]. Other commonly used PROMs include Visual-Analogue-Scale Foot and Ankle (VAS-FA) [52] (also validated in the Finnish language [53]), Visual-Analogue-Scale (VAS) [28], Short Form 36 (SF-36) [28, 33], Baltimore Painful Foot Score (PFS) [31], Short Musculoskeletal Function Assessment (SMFA) [33], long-form Musculoskeletal Function Assessment (MFA) [13], the Maryland foot score [47] and activities of daily living (ADL) [47]. In our study, we decided to use AOFAS because it is the most commonly used PROM for Lisfranc injuries and VAS-FA as it is validated in the Finnish language [53].

### Previous studies

The literature does not provide any prospective randomized controlled studies on the nonoperative versus the operative treatment of Lisfranc injuries. Current knowledge is based on a few case-series [35, 37] and retrospective studies [5, 22, 38]. Nunley and Vertullo [29] suggested in their series of midfoot sprains in athletes that only totally non-dislocated sprain injuries should be treated nonoperatively, and that all injuries where the diastasis between the first and second metatarsal is 2 mm or more would benefit from ORIF. Myerson et al. [5] were the first to study the nonoperative treatment of Lisfranc injury. In their study, only 5 out of a total of 52 patients were treated nonoperatively, and these patients received the treatment unintentionally, due to incorrect diagnosis. Of these five patients, four resulted in a poor result and one resulted in a fair result. Curtis et al. [22] organized a retrospective study of the treatment of 19 athletes with Lisfranc injuries. Only 14 stable injuries were treated nonoperatively. An excellent functional result was obtained with six patients, a good result with three patients, a fair result in four and a poor result with one patient. An excellent result implied the absence of symptoms and signs; a good result implied minor symptoms or signs; a fair result implied residual signs of symptoms with some disability, and a poor result implied marked symptoms or signs with limitation of function and a request for further treatment, such as arthrodesis. The treatment protocol between patients differed from “none” to “cast for ten weeks”. Crates et al. [38] studied nonoperative treatment and operative treatment after the failed nonoperative treatment of subtle Lisfranc injuries in 36 patients. The nonoperative protocol consisted of 6 weeks of a short leg walking orthosis and weight-bearing was progressed as tolerated. Progressed weight-bearing in an orthotic was begun after boot removal. Nonoperative treatment was successful in 16 patients, and the treatment failed in 20 patients. The mean AOFAS midfoot score in the successfully treated patients was 62 (49–72) before treatment and 75 (53–100) after treatment.

There have only been two previous prospective randomized studies on ORIF vs PA. Ly and Coetzee [30] randomly assigned 41 patients with ligamentous Lisfranc injuries to either an ORIF group or a PA group. The PA group had a slightly better functional outcome (AOFAS score 88 vs. 69), a higher return to preinjury activity level (92% vs. 65%), a lower rate of revision surgery and less pain in the final follow-up. Implant removal due to prominent or painful screws was performed on 16 of the 20 patients in the ORIF group and on 4 of the 21 patients in the PA group. The implant removal was only performed due to painful hardware, on average at 6.5 months (range: from five to ten months). Follow-up radiographs showed loss of correction, increasing deformity, and degenerative joint disease in 15 of the 20 patients in the ORIF group and 7 of them required conversion to an arthrodesis. In the PA group, one patient had delayed union at seventeen weeks and one patient required a revision arthrodesis with bone graft. One patient suffered from a post-traumatic intrinsic compartment syndrome that resulted in claw toes. In the study by Henning et al. [33], 40 patients with acute Lisfranc joint fractures or fracture dislocations were randomized to primary ORIF or PA. A total of 8 patients dropped out before 3-months follow-up. There was a significantly higher rate of secondary surgery in the ORIF group. Statistically significant differences were not found in physical functioning with regard to SF-36 or SMFA scores at any follow-up time interval. In their systematic review and meta-analysis, Smith et al. [34] concluded that ORIF has a higher risk of implant removal compared with PA (risk ratio 0.23 (0.11–0.45) *p* < 0.001), although there were no statistically significant differences in revision surgery, PROMs or non-anatomic alignment. Cochran et al. [32] organized a retrospective comparative cohort study on PA versus ORIF in young athletic military personnel with low-energy Lisfranc injury. In their study, PA resulted in a faster return to military service, a lower implant removal rate and better fitness scores after 1 year.

In conclusion, PA seems to result in less secondary surgery, less implant removal and a faster return to activity. There is some evidence of a better functional outcome after arthrodesis, but the result is still controversial. Nevertheless, the current overall evidence slightly favors arthrodesis as a primary treatment of dislocated Lisfranc injuries.

### Aims of this study

The aim of this two-armed randomized controlled trial is to I) compare nonoperative treatment with ORIF in non-dislocated Lisfranc injuries and II) to compare ORIF with PA in dislocated Lisfranc injuries.

## Methods/design

The study is a prospective, randomized, national multicenter trial. The trial centers are Tampere University Hospital and Seinäjoki Central Hospital. The trial has been designed to compare the nonoperative and operative treatment of Lisfranc injuries. The trial includes two strata: Stratum I compares nonoperative treatment and operative treatment with ORIF for non-dislocated Lisfranc injuries. Stratum II compares ORIF and PA in dislocated Lisfranc injuries.

The primary outcome in this study is the AOFAS [54] measured after 6, 12 and 24 months. The secondary measured outcomes after 6, 12 and 24 months are VAS [55], VAS-FA [52], number of secondary operations (implant removal, secondary arthrodesis) and number of other treatment-related complications.

### Hypotheses

Our primary hypotheses in the study are the following:

i) The hypothesis of stratum I is that nonoperative treatment of non-dislocated Lisfranc injuries yields better outcome in terms of AOFAS, VAS and VAS-FA score compared with operative treatment (ORIF).
ii) The hypothesis of stratum II is that PA of dislocated Lisfranc injuries yields better functional outcome in terms of AOFAS, VAS and VAS-FA score compared with ORIF, and PA results in fewer secondary operations than ORIF.

The results of both strata will be analyzed and reported separately.

### Patient selection and methods

**Fig. 1:**
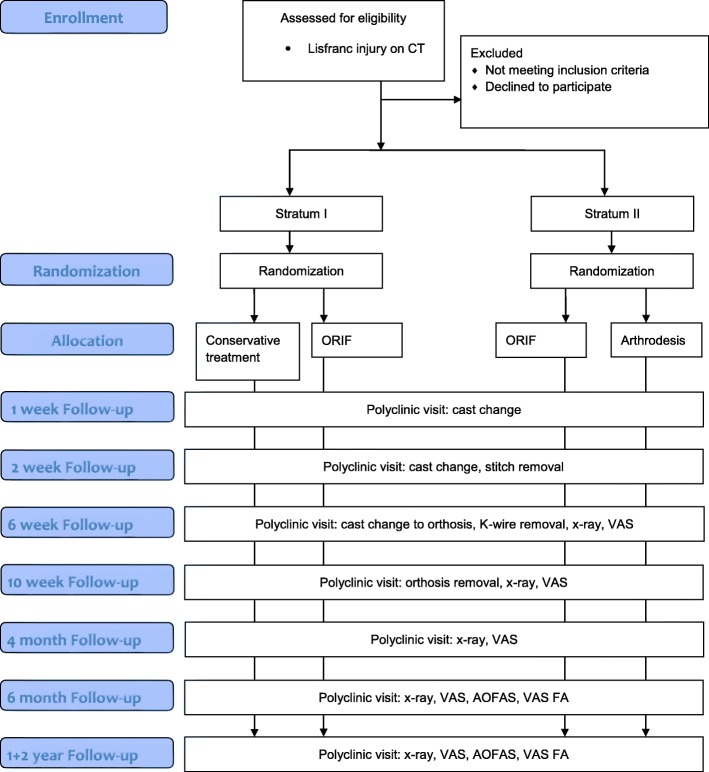
Flow diagram

The study population comprises patients suffering from acute Lisfranc joint injury (Fig. 1). Clinical suspicion (pain, swelling, plantar ecchymosis or gap sign) or typical findings on plain x-ray (‘fleck sign’, avulsion or fracture) leads to CT where the diagnosis and morphology of the injury is confirmed. Eligible patients are informed about the study at the emergency room (ER) by the surgeon on call. The final eligibility of patients and correct study strata is determined based on CT findings and other medical information and discussion with the patient by one of the foot and ankle surgeons in the study group (HH, H-JL, HMM, JJ, OV).

### Inclusion criteria

Stratum I (nonoperative treatment vs. ORIF):

- Non-dislocated (< 2 mm) fractures affecting TMT joints II and III
- And/or Dislocation < 5 mm between medial cuneiform and base of MT II
- And no fractures affecting TMT joints IV and V

Stratum 2 (ORIF vs. PA):

- Affected joints TMT II - III + any other TMT
- Any dislocation > 2 mm (fracture or TMT joint)
- Dislocation > 5 mm between medial cuneiform and base of MT II

### Exclusion criteria

- Aged under 18 or over 60
- Open fractures
- Extra-articular metatarsal fractures
- Extremely comminuted fractures with bone loss and poor chance of gaining proper fixation with screws
- Polytrauma patients
- Patients with weak co-operation (dementia, alcohol use, etc.)
- Patients with significant neuropathy or some other neurological condition
- Diabetes
- Rheumatoid arthritis
- Patients with severe circulatory disorder of the lower limb
- A delay in diagnosis of more than 14 days
- Patients with a previous foot injury or surgery of the injured foot
- Pregnancy
- Patients who refuse to participate

### Randomization

All patients will be randomized by the research coordinator at Tampere University Hospital who will not participate in the study. Patients with non-dislocated injuries are randomized into a nonoperative or ORIF group. Patients with dislocated injuries will be randomized into ORIF or PA groups. Both injury types will be randomized in blocks of ten. The treatment allocations from the randomization will be sealed in envelopes which will be then used and opened in numerical order after patient enrolment has been confirmed by the research physician. The research coordinator will monitor the study flow.

### Nonoperative treatment

Nonoperative treatment is conducted with non-weight-bearing cast-immobilization for 6 weeks. The cast is changed at 1 and 2 week controls. The cast is removed at 6 weeks and patients are prescribed a walking boot for 4 weeks. Weight-bearing with a walking boot is limited to half-bodyweight for the first 2 weeks and the last 2 weeks as tolerated. At 10 weeks, patients will be allowed to use their own shoes and walk as tolerated.

### Surgical technique

The surgical procedures will be performed by experienced foot and ankle surgeons (HH, H-JL, HM, JJ and OV). All patients will receive an antibiotic prophylaxis preoperatively. The operation is performed under tourniquet at 280 mmHg to 300 mmHg pressure.

### Open reduction and internal fixation

**Fig. 2:**
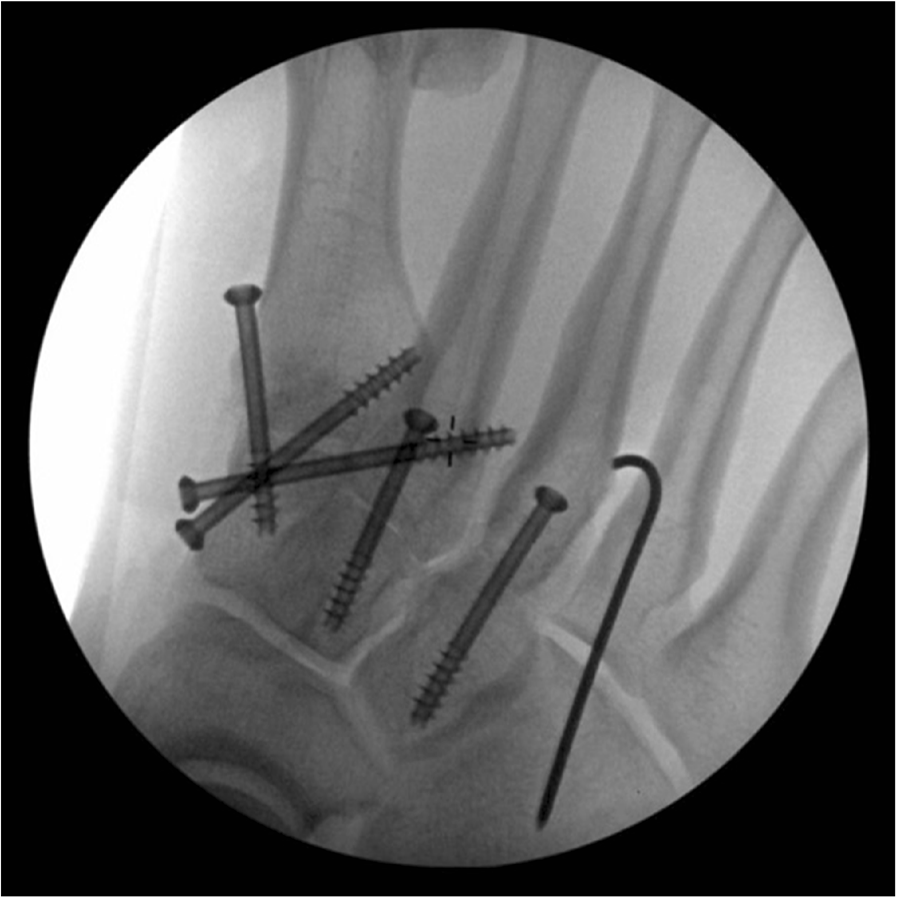
Intraoperative view of the screw and K-wire fixation of the TMT joints

One or two incisions will be made depending on the location of the injury. Only the affected and instable TMT joints are fixed. The first incision is made between MT I-II and the second incision (if necessary) at the base of MT IV. Open anatomical reduction and screw fixation of the 2nd metatarsal to the medial cuneiform bone (‘home run screw’) and affected TMT joints will be performed with 4.0 cannulated screws (DePuySynthes©, Stryker©). If TMT IV or V joints are dislocated, after open reduction of those joints, temporary fixation with Kirschner-wires will be used (Fig. 2). Fixation will be performed under fluoroscopic guidance. K-wires will be cut, bent and left visible on the skin and removed at the 6 week postoperative visit. Wounds will be closed with dermal sutures. Fixation screws will be removed only if they cause any symptoms.

### Primary arthrodesis

Incisions will be made as described for ORIF. Cartilage and fibrous tissue will be removed from the affected TMT joints with a chisel. Fixation for the medial cuneiform bone to the base of 2nd metatarsal and TMT I-III will be performed with 4.0 cannulated screws in a similar manner to ORIF. If TMT IV or V joints are affected, arthrodesis will not be done, but temporary fixation will be performed, as described for ORIF.

### Postoperative aftercare

Postoperative aftercare is identical to nonoperative treatment with 6 weeks of non-weight-bearing cast-immobilization and 4 weeks of walking boot. Stitches are removed, and cast changed at 2-week visit. The cast and K-wires stabilizing the TMT IV and/or V joints are removed at 6-week visit. Thrombosis prophylaxis and analgesic medication is planned individually.

### Follow-up

All follow-up visits will be conducted in the trauma outpatient clinic of the hospital where the patient was primarily treated (Table 1). The visits are at 6 weeks, 10 weeks, 6 months, 12 months and 24 months after the injury. Standing x-ray of the injured foot and VAS score is obtained during every visit. AOFAS and VAS Foot and Ankle questionnaires will be completed during the 6, 12 and 24-month visits.

**Table 1:** Assessments and procedures of the trial

	Medical history	Radiograph	CT	VAS	AOFAS	VAS FA
Baseline	X	X	X			
6 weeks		X		X		
10 weeks		X		X		
4 months		X		X		
6 months		X		X	X	X
1 year		X		X	X	X
2 years		X		X	X	X

### Power analysis

In this trial, the widely recognized AOFAS will be used as the main outcome measure. The clinically significant difference in AOFAS has been reported to be 8.36 (SD 11.16) points [56]. Assuming a 10-point difference in the AOFAS score and a standard deviation of 12 points, the estimated sample size is 23 patients (delta = 10, sd = 12, alpha = 0.05, power 0.8). We will assume a 20% drop-out rate in both groups, and therefore the total patient count needed for both stratums will be 56 patients. Due to block randomization in blocks of ten, 60 patients will be recruited.

### Statistical analysis

The baseline characteristics will be reported as mean (standard deviation), median (quartiles) or proportion. Study groups will be compared using t-test, Mann-Whitney U or Fisher’s exact test. Primary (AOFAS) and secondary outcomes (VAS-FA, VAS, complications, secondary surgery) will be compared at 12 months and 24 months using the Mann-Whitney U test. The results will be presented with 95% confidence intervals, and therefore a *p*-value of < 0.05 will be considered statistically significant. The data will be analyzed according to the intention-to-treat principle, assuming the patients change group during the study. The statistical analysis will be performed with SPSS© version 22.

### Study material

All information will be sent to Tampere University Hospital and the gathered material will be stored in a study registry. The registry is protected with passwords given only to the authors and the secretary of the study group and the data will be deleted 15 years after the end of the study.

### Ethics

The study protocol and additional papers, including consent form, patient information form and questionnaires have been approved by the Regional Ethics Committee of Tampere University Hospital**.** (Approval number R11152, 11th November 2011). All participants will provide a written consent to participate.

### Time schedule

The recruitment of patients started in 2011 and it will be continued until the number of patients achieves the estimated volume of power analysis. The final results will be analyzed after the 2-year follow-up period of the last recruited patient. In October 2017, 51 patients had been included in the study. The final report will be published by the end of 2021.

## Discussion

This publication presents a prospective, randomized, national multi-center trial. It gives details of patient flow, randomization, aftercare and methods of analysis of the material and ways to present and publish the results. The limitations of this study are limited patient blinding due to the nature of the treatment (operative versus nonoperative) and using a primary outcome measure (AOFAS) that has not been validated in Finnish. The strength of this study is that this is the first study to compare the nonoperative and operative treatment of Lisfranc joint injuries in a prospective and randomized study setting with an adequate number of patients. As the previous literature provides only two contradictory randomized controlled trials on this matter, the benefits of this study are to provide evidence-based knowledge on the treatment of these injuries.

## Abbreviations

ADL: Activities of daily living
AOFAS: American Orthopaedic Foot and Ankle Society
CT: Computed tomography
ER: Emergency room
K-Wire: Kirschner-Wire
MFA: Long-Form Musculoskeletal Function Assessment
MT: Metatarsal
ORIF: Open reduction and internal fixation
PA: Primary arthrodesis
PFS: Baltimore Painful Foot Score
SF-36: Short-Form 36
SMFA: Short-Form Musculoskeletal Function Assessment
TMT: Tarsometatarsal
VAS FA: Foot and Ankle Visual-Analogue-Scale
VAS: Visual-Analogue-Scale
